# Defining the Role of Immunotherapy in the Curative Treatment of Locoregionally Advanced Head and Neck Cancer: Promises, Challenges, and Opportunities

**DOI:** 10.3389/fonc.2021.738626

**Published:** 2021-09-21

**Authors:** Robert Saddawi-Konefka, Aaron B. Simon, Whitney Sumner, Andrew Sharabi, Loren K. Mell, Ezra E. W. Cohen

**Affiliations:** ^1^Department of Surgery, Division of Otolaryngology-Head and Neck Surgery, UC San Diego School of Medicine, San Diego, CA, United States; ^2^Moores Cancer Center, UC San Diego, La Jolla, CA, United States; ^3^Department of Radiation Oncology, UC Irvine School of Medicine, Irvine, CA, United States; ^4^Department of Radiation Medicine and Applied Sciences, UC San Diego School of Medicine, San Diego, CA, United States; ^5^Department of Medicine, Division of Hematology-Oncology, UC San Diego School of Medicine, San Diego, CA, United States

**Keywords:** immunotherapy, head and neck (H&N) cancer, curative treatment, immune oncology (IO), treatment sequences

## Abstract

Recent advancements in the development of immunotherapies have raised the hope for patients with locally-advanced HNSCC (LA-HNSCC) to achieve improved oncologic outcomes without the heavy burden of treatment-related morbidity. While there are several ongoing late phase clinical trials that seek to determine whether immunotherapy can be effectively employed in the definitive setting, initial results from concurrent immuno-radiotherapy therapy trials have not shown strong evidence of benefit. Encouragingly, evidence from preclinical studies and early-phase neoadjuvant studies have begun to show potential pathways forward, with therapeutic combinations and sequences that intentionally spare tumor draining lymphatics in order to maximize the synergy between definitive local therapy and immunotherapy. The intent of this review is to summarize the scientific rationale and current clinical evidence for employing immunotherapy for LA-HNSCC as well as the ongoing efforts and challenges to determine how to optimally deliver and sequence immunotherapy alongside traditional therapeutics. In both the preclinical and clinical settings, we will discuss the application of immunotherapies to both surgical and radiotherapeutic management of HNSCC.

## Introduction

Squamous cell carcinomas arising from the upper aerodigestive tract, including the pharynx, larynx, and oral cavity, present unique therapeutic challenges in oncology. Representing approximately 3% of new cancer cases and 3% of cancer deaths worldwide (1), head and neck squamous cell carcinomas (HNSCC) arise adjacent to or within anatomy central to ventilation, speech, mastication, salivation, and swallowing. As such, progression of HNSCC causes significant morbidity, even in the non-metastatic setting. Similarly, curative-intent treatments for HNSCC, which also compromise aerodigestive function, carry a high degree of morbidity. In select cases of early-stage disease, surgical extirpation alone may be sufficient; however, in cases of locally advanced disease, curative-intent treatment often requires multimodal therapy with surgery, radiation therapy, and chemotherapy. The sequelae of curative-intent treatment are manifold and may include permanent alterations in swallowing function, speech, taste, salivation, and dental health. At the same time, even with aggressive therapy, loco-regional and distant recurrences after treatment are all too common and carry a dismal prognosis (2).

While innovations in both surgical technique (3) and radiation therapy (4) have led to improvements in treatment related morbidity without sacrificing treatment efficacy, there remains a critical need for therapies that can deliver cures without incurring undue toxicity (5). Two decades ago, the addition of cisplatin chemotherapy to definitive radiation improved oncologic outcomes, albeit at the cost of significant additional toxicity (6–8). The advent of molecularly targeted therapies in the 1990s raised hopes that cisplatin chemotherapy could be replaced by less toxic targeted systemic therapies, particularly after the EGFR-inhibitor cetuximab added to radiotherapy was shown to improve outcomes over radiation therapy alone (9). Unfortunately, several recent studies have shown inferior outcomes with cetuximab *versus* cisplatin given concurrently with radiation (10, 11) and newer targeted agents are yet to demonstrate comparable efficacy in clinical trials (12). However, the recent development and clinical implementation of novel immunotherapeutic agents - drugs that promise to overcome tumor-immune evasion and stimulate an anti-neoplastic immune response - has once again raised hopes that the efficacy of treatment for HNSCC can be enhanced without increasing treatment-related morbidity.

## Preclinical Models

Immunotherapies represent a unique class of cancer therapeutics that target host immunity to invigorate systemic anticancer immunity. This therapeutic strategy diverges from traditional therapies which target cancer cells specifically to induce cytotoxicity or inhibit growth. It is precisely for this reason that the field has had to refocus overall therapeutic algorithms. Moreover, because the anti-tumor effects of immunotherapy depend on interactions amongst multiple organ systems (hematologic, lymphatic, vascular), it is critical that we study these therapies in immunocompetent, syngeneic *in vivo* model systems, a requirement that has brought with it new methodological challenges.

Preclinical models of murine HNSCC include *ex vivo* models derived from human disease and primary murine models arising either spontaneously after carcinogen exposure or driven by activated oncogenic signaling networks. The variety of contemporary preclinical models reflects their utility and relevance to address open questions in the field, with each model offering certain advantages and limitations.

### *Ex Vivo* Derived Preclinical Models

Human Derived tumor models have played a critical role in the study of traditional chemotherapies and targeted therapies. However, by virtue of their inherent immunogenicity in animal models, they are limited in their ability to inform immunotherapy research. Immortalization of *ex vivo* cultured HNSCC cells was first reported four decades ago (13). Since then, several groups have rigorously profiled panels of the more commonly employed immortalized HNSCC lines, culminating in the assembly of repositories such as the Cancer Cell Line Encyclopedia (CCLE) (14, 15). Collectively, these efforts inform our understanding of the genetic and molecular drivers of HNSCC and serve as an ideal platform for novel therapeutic testing (16). Additionally, they afford insight into the extrinsic selective pressures imposed by antitumor immunosurveillance that shape tumor heterogeneity and clonal selection (17), a phenomenon predicted by the immunoediting hypothesis (18, 19).

Similar to immortalized cell line models, organoid and related three-dimensional models derive directly from human tissues. As first documented in a pharyngeal mucosa-derived organoid model (20), these models feature the advantage of a more physiologically representative tissue architecture and cellular milieu. Interestingly, divergent response profiles have been observed between three- *versus* two-dimensional patient derived models (21–23) with three-dimensional systems modeling the clinical responses to radiation more closely (24, 25). Three-dimensional tissue culture systems also offer the ability to deconstruct the proximal events in tumorigenesis. This was elegantly demonstrated in a series of studies identifying the central role of cancer-associated fibroblasts in tumorigenesis of the lingual mucosa (26, 27). More recently, in an effort to recapitulate the dynamic tumor-immune microenvironment *ex vivo*, Neal et al. developed the unique air-liquid-interface model for both murine-derived and patient-derived organoids (28). With this unique model system, the authors demonstrated a faithful representation of not only neoplastic cells and the endogenous immune infiltrate, including the complete tumor infiltrating lymphocyte repertoire, but also the native responsiveness to immune checkpoint inhibition (ICI) in organoids from > 100 human biopsies and murine tumors.

Patient-derived xenografts (PDXs) comprise a unique preclinical modelling strategy in which fragments of patient tumors are directly implanted into animals. PDXs are well-suited for targeted drug screens and examination of oncogenic signaling. However, use of this model *in vivo* is necessarily limited to immune-deficient animals or in humanized rodent models as PDXs are inherently immunogenic and are rejected in immune competent animals. Generally, humanized preclinical models are generated by inoculating immune-deficient recipient animals with either (i) human peripheral blood mononuclear cells; (ii) human CD34+ hematopoietic stem cells (HSCs); or, (iii) concurrent human CD34+ HSCs and autologous human fetal liver and thymus tissue transfer. While humanized models offer tremendous opportunity to study PDXs-immune system interactions *in vivo*, they are limited primarily by graft *versus* host disease in recipient animals and by human donor variability. For a more detailed information regarding contemporary HNSCC PDXs and human preclinical modelling see (29–33).

### Primary Murine Preclinical Models

In contrast, syngeneic tumor models, which are derived from inbred mice and can be transplanted into immune-competent animals of the same strain, have afforded tremendous insight into mechanisms of tumor-immune evasion and immunotherapeutic resistance. Syngeneic models include those that have arisen spontaneously or as a consequence of carcinogen exposure. The SCC VII/SF model, which was derived from a spontaneous cancer in C3H/HeJ mice, is among the most widespread, contemporary spontaneous, syngeneic HNSCC models (34). Another versatile and popular model is the panel of murine oral-cavity (MOC) squamous cell lines developed with exposure of 7,12-dimethylbenz(a)anthracene (DMBA)-induced (35, 36). Early therapeutic efforts with these models focused on delivery of systemic immunotherapy with stimulatory cytokine delivery (37, 38). More recently, syngeneic models have served as the mainstay to evaluate advancements in immunotherapy with checkpoint blockade inhibitors and adoptive cell transfer.

Arguably, the ideal preclinical HNSCCC model is one which mimics the oncogenic mutanome of its human disease counterpart, can be transplanted orthotopically, progresses in immune-competent hosts and features both an immune infiltrate and a response to immunotherapy similar to that observed clinically. Lastly, the ideal preclinical model should be dynamic both in its genesis and behavior *in vivo*; and, as such, reflect more accurately the dynamic changes occurring downstream from selective immune pressure, as has been documented in human disease over time (17, 39). Collectively, these properties offer the greatest promise to not only better understand fundamental cancer-immune dynamics but also develop translatable therapeutic strategies and biomarkers of therapy response.

Excitingly, examples of such ‘next-generation’ preclinical models are emerging. Following from early 4NQO-carcinogen-induced models (40, 41), newer syngeneic, orthotopic murine oral SCC model demonstrate a remarkable homology to the human tobacco-signature mutanome (42, 43). Such models are ideally suited to efforts aimed at understanding tumor-immune interactions and, by extension, developing precision immune-oncology therapies, as described below.

A parallel effort in preclinical HNSCC modeling is to manufacture carcinogenesis by driving specific oncogenic pathways, and, in so doing, generate genetically engineered mouse models (GEMMs). Historically, preclinical GEMMs were derived by driving oncogenic programs downstream from K-ras activation (44–47). However, based upon several studies of common mutational signatures amongst human cancers (48–50), early GEMMs have come under scrutiny for featuring genetic alterations rarely seen in HNSCC and lacking the signature mutanomes now associated with both human papillomavirus (HPV)-positive and HPV-negative HNSCC (49, 50). However, efforts aimed at deconstructing oncogenic networks in HNSCC tumorigenesis are particularly-well suited to the GEMM preclinical platform. GEMMS affords an opportunity to examine the key milieu of true genetic drivers with the promise of exposing targetable molecular vulnerabilities in HNSCC (51). These efforts are especially true with regards to modeling HPV-associated disease in which targeting viral E6/E7 expression in turn activates genome-wide oncogenic changes (52).

## Preclinical Immunotherapeutic Strategies

Cancer-immune dynamics underlie the efficacy of IO therapies. While major advancements in the management of HNSCC have been achieved by examining immune checkpoint inhibition in the clinic (53, 54), innovation in IO therapy design is tempered by an incomplete understanding of cancer-immune interactions, as evidenced by unexpectedly equivocal results in several recent clinical trials (55–58).

The advent of clinically-relevant, robust preclinical models addresses this problem by affording the opportunity to scrutinize not only anti-tumor immunity but also cancer-derived influence over host immunity. A testament to the power of translating preclinical observations from clinically-relevant models is the breadth of emerging clinical trials examining immune-oncology therapies in HNSCC (42, 59). The following subsections highlight only a fraction of the ground-breaking preclinical work that have contributed to the advancement of IO-therapy design and our collective understanding of cancer-immune interactions in HNSCC.

Because the efficacy of immunotherapies depends on their interactions with tumor, hematologic, and lymphatic organ systems, in the preclinical realm, there is considerable interest in understanding how traditional anti-cancer therapies, surgery, radiation, and systemic therapies affect these interactions and modulate the effects of immunotherapies. There is considerable work underway to optimize the interactions between surgery, radiation, and other systemic therapies and immunotherapies in order to maximize synergy and minimize interference.

### Radiation Therapy and Immunotherapy

Combinations of radiation therapy and immunotherapies have been increasingly explored both in the preclinical and clinical arenas (42, 60, 61). A question of considerable interest in the field is whether radiation dose and fractionation can be optimized in order to maximize the synergistic effects of immunotherapy and radiation therapy. Specifically, there is increasing interest in determining whether hypofractionation of radiotherapy delivery (delivery of larger daily radiation doses over a shorter time period, typically to a lower total dose) can improve the anti-neoplastic immune response stimulated by modern immunotherapeutic agents. The rationale for this approach is based in the theory that radiation therapy not only kills cancer cells through direct damage to their DNA, but also *via* stimulation of antitumoral immunity (62). However, radiation therapy can also have detrimental effects on the tumor-immune environment, including toxic effects on the local immune cell population and in secondary lymphoid organs (61). Thus, a careful balance between the immunostimulatory and immunosuppressive effects of radiation therapy must be achieved to optimize synergy between immunotherapies and radiotherapy.

There is growing preclinical evidence across multiple cancer histologies that hypo-fractionated radiation better achieves this balance than conventionally fractionated radiation. In their seminal 2009 paper, Lee et al. demonstrated that the antitumor effects of highly hypo-fractionated radiation (20Gy in 1 fraction) on B16 melanoma tumors was significantly greater in wild type mice than immune-incompetent nude mice (63), suggesting that the immune system mediates a portion of radiation therapy’s antineoplastic effects. Further, they found that by dividing the delivered radiation dose over 4 days (20Gy in 5 fractions), there was a loss of tumor control that was similar to the effect of treating wild type mice with an anti-CD8 antibody after single fraction treatment. They found that highly hypo-fractionated radiation significantly increased dendritic cell maturation and promoted priming of antigen-specific T cells, and they hypothesized that hypo-fractionated radiation might have greater synergistic effects with immunotherapy than conventionally fractionated radiation. Subsequently, Grapin et al. investigated the effects of different radiotherapy schedules with equivalent biologically effective doses on the intra-tumoral immune response of mice bearing subcutaneous CT26 colon tumors (64). They found that while conventionally fractionated radiation (36Gy in 18 fractions) induced a myeloid response dominated by myeloid derived suppressor cells (MDSC) and type 2 tumor associated macrophages (TAM 2), highly hypo-fractionated radiation (either 16.4Gy in 1 fraction or 24Gy in 3 fractions) induced a lymphoid response dominated by CD8+ T-cells and regulatory T cells. Delivery of 24Gy in 3 fractions was also found to induce the highest proportion of T cells secreting granzyme B, while conventional fractionation was found to induce the most durable increase in tumoral expression of PD-L1. Similarly, Lan et al. found in mice bearing subcutaneous LL/2 lung tumors or B16F10 melanoma tumors that highly hypo-fractionated radiation to 23Gy in 2 fractions reduced recruitment of MDSCs into the tumor microenvironment and decreased PD-L1 expression compared with more modestly hypo-fractionated radiation to 36Gy in 9 fractions (65). Together, this growing body of preclinical work suggests that hypo-fractionated radiation is more likely to stimulate an antineoplastic immune response *versus* conventionally fractionated radiation although this remains to be demonstrated in randomized human studies.

### Surgery and Immunotherapy

There is also mounting preclinical evidence in support of the employment of immunotherapeutic agents in the neoadjuvant setting prior to definitive surgical intervention. Preclinical models suggest that the efficacy of checkpoint inhibitor therapy may be dependent on communication between the primary tumor and its regional draining lymph nodes (66). Tumor draining lymph nodes have long been implicated in the presentation of tumor antigen to and activation of cytotoxic T-cells in response to tumor growth (67), and enhancement of antigen presentation in lymph nodes through expansion and activation of intratumoral dendritic cells has been shown to potentiate the response to checkpoint inhibition in pre-clinical models. More recently, Liu et al. found improved survival in a mouse model of triple negative breast cancer when checkpoint inhibition was initiated prior to rather than after surgical resection of the primary tumor (68). The improvement in efficacy was associated with a significantly greater increase in tumor-specific CD8 T cells in the peripheral blood and organs of mice treated with neoadjuvant *vs*. adjuvant immunotherapy, suggesting a more robust immune response in the setting of an intact primary tumor. Similarly, Fransen et al. found in two murine orthotopic colon cancer models that the antineoplastic response to checkpoint inhibition was dependent on intact communication between the tumor and draining, but not distant, lymphatics (69). Surgically excising the draining lymph nodes associated with flank tumors significantly diminished the effects of subsequently administered anti-PD-1 therapy. No reduction of efficacy was observed when the contralateral non-draining lymph nodes were excised prior to anti-PD-1 administration. Reduced efficacy was also demonstrated when an S1P receptor inhibitor, which blocks T cells egress from lymphoid organs, was administered prior to anti-PD-1 therapy, suggesting that the effects of the checkpoint inhibitor were mediated by activation of T cells within the regional lymphatics. Taken together, this growing body of preclinical work provides strong rationale for clinical study of checkpoint inhibition in the neoadjuvant setting, when communication between the primary tumor and its draining lymphatics has not yet been disrupted either by surgical excision or radiation.

### Systemic Therapy and Immunotherapy

Similarly, a growing body of literature now indicate that systemic therapies (chemotherapies and targeted therapies) can potentiate immunotherapy, either by inducing immunogenic tumor cell death with antigen shedding (70–72) or through depletion of immunosuppressive effector populations within the tumor microenvironment (73–76).

Collectively, observations gleaned from parallel preclinical investigations have converged upon certain key mediators that regulate the response to immunotherapy in HNSCC (76, 77). An illustrative example of such a convergence point in HNSCC that has progressed from the preclinical to clinical arena is the myeloid derived suppressive cell (MDSC). MDSCs are notorious for their role in suppressing antigen-specific T cell cytotoxicity and mediating acquired resistance to immunotherapy (78, 79). In preclinical models of HNSCC, MDSCs have specifically been found to regulate the response to PD-1 checkpoint blockade therapy (80, 81). Interestingly, independent groups have found that selective targeting of the PI3K signaling axis can reverse MDSC accumulation by altering tumor-derived cytokine production, ultimately leading to improved PD-1 responses. These key insights suggest that oncogenic signaling in HNSCC is intrinsically linked to maintaining an immunosuppressive tumor immune microenvironment (TIME). In tandem, other investigators, leveraging the power of next generation preclinical models, have innovated to identify oncogenic aberrations upstream from PI3K signaling as an exploitable and precision therapy target to combine with PD-1 blockade in a similar fashion (42). These advancements in fundamental cancer-immune interactions in HNSCC open the door for paradigm-altering treatment strategies that combine targeted molecular cancer therapies with immunotherapies.

## Clinical Evidence Supporting Use of Immunotherapy in LA-HNSCC

### Concurrently With Radiation

Concurrent chemoradiation, with conventionally fractionated radiation and cisplatin-based chemotherapy, is the standard of care definitive treatment strategy for inoperable LA-HNSCC (82). While employment of chemoradiation has produced significant improvements in outcomes compared with radiotherapy alone, it also can result in significant treatment-related morbidity and outcomes remain poor for many patients. Efforts to replace or augment cisplatin-based chemoradiation with targeted systemic therapies have to date produced disappointing results in the clinical setting (10, 11, 83, 84). In contrast, by virtue of their demonstrated efficacy in the recurrent and metastatic setting, immunotherapies present a promising alternative (54, 85, 86). A great deal of work in this area is ongoing, and early results from the initial clinical trials have not been uniformly encouraging (57). However, even negative studies yield important insights into how best to employ immunotherapy with definitive radiotherapy.

**Table 1** highlights an illustrative listing of recent efforts to interrogate the efficacy of combining IO and radiotherapy. Among these initial efforts, Powell et al. conducted a phase IB study evaluating the safety and efficacy of pembrolizumab when given during and after definitive radiation therapy with concurrent weekly cisplatin (87). The study enrolled 59 patients with locally advanced (Stage III-IVB, AJCC 7^th^) HNSCC regardless of HPV status with the majority (67.8%) presenting with oropharyngeal primaries. Safety was the primary endpoint of the study with efficacy assessed by the rate of complete response as defined by imaging or pathologic criteria. The therapy was overall well tolerated with only 5 patients (8.8%) requiring discontinuation of pembrolizumab due to toxicity and all but one patient receiving the full planned dose of radiotherapy without any treatment delays exceeding five days. The complete response rate (CR) was 85.3% and 78.3% among HPV-positive and HPV-negative patients, respectively, which met the pre-specified response in the HPV-negative but not HPV-positive cohorts. Progression free and overall survival outcomes were encouraging, with 2-year overall survival (OS) and progression-free survival (PFS) of 86.5% and 72.6%, respectively, for the HPV-negative cohort and 97.1% and 92.8%, respectively, for the HPV-positive cohort. This study concluded that pembrolizumab given concurrently with cisplatin based chemoradiation was safe and did not limit delivery of definitive chemoradiation.

**Table 1:** Illustrative listing of clinical trials examining combination immunotherapy and definitive radiation for LA-HNSCC.

Trial	Ref	Phase	Study Design	Study Eligibility	Primary Outcome	Status	Estimated Completion
NCT02586207	Powell JCO 2020	IB	Pembro (200mg d-7) → CRT → RAA + Pembro (200mg x5)	LA-HNSCC (Stage III-IVB)	Safety/Tolerability	Active, Not Reruiting	Sep-23
NCT02952586	Lee Lancet 2021	III	Avelumab (10mg/kg x1, d-7) → Avelumab (10mg/kg q2wk) + CRT → RAA + Avelumab (10mg/kg q2wk)	LA-HNSCC	PFS	Discontinued	Aug-20
NCT03383094	n/a	III	Pembro (200mg q3wk x20) + RT	LA-HNSCC	PFS	Recruiting	Jun-24
NCT03258554	n/a	II/III	Durva (1500mg wk-2 x7) + RT	LA-HNSCC	DLT/PFS/OS	Recruiting	Dec-25
NCT02707588	Bourhis AnnOnc 2020	II	Pembro (200mg q3wk) + RT	LA-HNSCC	LRC	Active, Not Reruiting	Dec-21
NCT03952585	n/a	II/III	Reduced-Dose RT + Cisplatin *vs* Nivo (d-7 q2wk x6)	LA-HNSCC	PFS/QOL	Recruiting	Feb-25

However, the promising efficacy results from the Powell study have not been confirmed by the more recently reported phase III trial, JAVELIN head and neck 100 (57). JAVELIN was a randomized, placebo-controlled, double blind trial in which patients with locally advanced HNSCC received chemoradiation with bolus cisplatin chemotherapy and either concurrent and adjuvant avelumab or placebo. The trial enrolled 697 patients and the primary endpoint was PFS. At interim analysis tolerability was similar in both arms. However, the trial was closed early after it was determined that PFS would not be improved in the experimental avelumab arm [hazard ratio (HR), 1.21; 95% confidence interval (CI) 0.93-1.57; 1-sided p = 0.920]. Similarly, OS was not improved in the avelumab arm [HR, 1.31; 95% confidence interval (CI) 0.93-1.85; 1-sided p = 0.94]. Subset analysis did not find any subgroup with improved outcomes on the avelumab arm, though there was a non-significant trend toward improved PFS in the patients with PD-L1 high tumors [HR 0.59, 95% confidence interval (CI) 0.28-1.22]. The failure of this highly anticipated trial to meet its primary endpoint raises a number of important questions. Given the demonstrated efficacy in the recurrent and metastatic setting, what could explain the failure of checkpoint inhibitors to improve outcomes in the upfront concurrent/adjuvant setting? The results of the study could not be easily explained by poor tolerability or increased toxicity with the study drug, as no significant differences in safety outcomes were observed in the investigational and placebo arms. Another potential explanation is that the efficacy of avelumab was restricted to patients with PD-L1 high tumors. While this may be the case, this potential subgroup effect in PD-L1 high expressing patients was not found to be statistically significant upon exploratory analysis in this study.

Alternatively, suboptimal IO treatment sequencing could be part of the reason underlying the negative results of the JAVELIN head and neck 100 study. The optimal timing of ICI relative to chemoradiation is unknown, and several trials conducted for non-HNSCC have shown benefit when employing ICIs in the adjuvant rather than concurrent setting. For example, the PACIFIC trial, a randomized, controlled phase III trial in unresectable locally advanced non-small-cell lung cancer (NSCLC), compared durvalumab consolidation therapy against placebo in patients with no disease progression after at least two cycles of platinum-based chemoradiotherapy, finding a significant benefit in progression-free and overall survival in the ICI group (PFS 16.8 months *versus* 5.6 month; HR 0.52, p<0.001; OS at 24 months 66.3% *versus* 55.6%, p=0.005) (88, 89). Similarly, the CheckMate 577 trial, a large, randomized, placebo-controlled phase III study evaluating ICI in the adjuvant setting in patients with esophageal or gastroesophageal junction cancer after neoadjuvant chemoradiation and surgery, found significant improvements in disease-free survival with ICI (22.4 months *versus* 11 months; HR 0.69, p<0.001) (90). These favorable results along with those from the CheckMate 141 (53) and KEYNOTE-048 trials (54) – both evaluating adjuvant ICI *versus* standard risk-adjusted chemoradiotherapy within the recurrent and metastatic HNSCC population – suggest that immune-oncology treatment sequencing may influence the tumor response to combination therapy.

A final, intriguing hypothesis is that the antineoplastic effects of avelumab were antagonized by the non-investigational components of the therapeutic regimen. One potential suspect is the cisplatin chemotherapy, given its known hematologic toxicity; however, this hypothesis is inconsistent with the results of the recent KEYNOTE-048 study – a large, randomized phase III trial in recurrent and metastatic HNSCC which found that ICI combined with platinum-based chemotherapy (including cisplatin) improved overall survival in the total study population (54). An alternative suspect is the radiation itself. As discussed above, conventionally fractionated radiation has been found to alter the tumor immune environment in ways that may antagonize immune surveillance and promote tumor-immune escape. Further, definitive intent radiation therapy entails elective radiation of the draining lymphatics of head and neck cancers, which may reduce the efficacy of immunotherapy by a similar mechanism to surgical lymphadenectomy.

A series of clinical studies that may shed additional light on these questions include those that assess whether checkpoint inhibitors can replace standard of care systemic agents in the setting of definitive intent radiation. Amongst these studies are the KEYCHAIN trial (NCT03383094) (91), the GORTEC 2015-01 ‘PembroRad’ trial (NCT02707588) (58), NRG HN004 (NCT03258554) (83) and NRG HN005 (NCT03952585). The KEYCHAIN trial is a prospective, multi-institutional, open-label, randomized phase II trial investigating whether concurrent and adjuvant pembrolizumab can improve PFS over standard concurrent cisplatin in patients with HPV-associated locally advanced HNSCC (Stage III-IVB, AJCC 8^th^) undergoing definitive intent radiation therapy. Both PembroRad and HN004 are prospective, randomized trials investigating whether checkpoint inhibitors (pembrolizumab and durvalumab, respectively) can improve outcomes over concurrent cetuximab when combined with definitive intent radiation therapy for cisplatin-ineligible patients. NRG HN005 is a prospective phase II/III trial interrogating progression-free survival and quality of life with reduced-dose definitive radiation with cisplatin or nivolumab compared to standard of care definitive chemoradiation. Of note, the PembroRad and HN005 trials omit adjuvant IO while the HN004 and KEYCHAIN trials include adjuvant IO to 3 months and 12 months, respectively. KEYCHAIN, HN004 and HN005 are actively enrolling at this time. PembroRad has completed enrollment and reported preliminary results at the ESMO 2020 annual conference (58). The trial enrolled 133 patients between 2016 and 2017 with a near-even split of p16-positive (46%) and p16-negative patients. Locoregional control at 15 months was 59% and 60% with cetuximab-RT and pembrolizumab-RT, respectively. There was no significant difference in two-year PFS. Notably, acute toxicity was lower in the pembrolizumab-RT arm compared to the cetuximab-RT arm, with at least one grade 3 or greater acute adverse event in 74% and 92% of patients, respectively. While the toxicity data reported in this trial is encouraging, the failure of pembrolizumab to improve PFS over cetuximab is disappointing, especially in light of the recent trials demonstrating the inferiority of cetuximab when compared with cisplatin. If KEYCHAIN and HN004 similarly fail to meet their efficacy endpoints, it may suggest a need to fundamentally rethink the timing, dose and fractionation, or nodal volume coverage of definitive-intent radiation therapy when delivered with checkpoint inhibitors.

While there is little clinical experience with elimination of elective nodal irradiation in the definitive setting, efforts have been made to study the effect of altered fractionation and dose. The current standard of care for definitive radiation therapy in LAHNSCC is to deliver 66-72Gy in approximately 2Gy daily fractions over 6-7 weeks to areas of gross disease, with modestly lower doses (44-63Gy) delivered to areas of subclinical disease, including elective nodal volumes, over the same time period. Compared to the burgeoning preclinical data, there is relatively little clinical evidence that highly hypo-fractionated radiation better stimulates a tumor immune response than conventionally fractionated radiation in patients with HNSCC. However, there is clinical evidence that modestly hypo-fractionated radiation can improve outcomes in certain clinical scenarios. For example, in early stage glottic larynx cancer, a prospective, randomized trial found that delivery of definitive radiation monotherapy in 2.5Gy fractions resulted in superior local control than delivery of a higher biologically effective dose (based on linear-quadratic modeling) in 2Gy fractions (92). At the time, the improved efficacy of the hypo-fractionated treatment regimen was attributed to the shorter overall treatment time needed to deliver the complete course of therapy, which may reduce the opportunity for cancer cells to repopulate during treatment. The results of this trial have led to the widespread clinical adoption of modestly hypo-fractionated radiation in early stage glottic larynx cancer. Outside of the glottic larynx, definitive radiation therapy continues to be standardly delivered in fractions of 2Gy or less; however, recently a retrospective case series of patients with oropharynx, hypopharynx, or larynx cancers treated at Princess Margaret from 2005-2017 has suggested that modestly hypo-fractionated radiation may be effective more broadly in the definitive setting (93). In this study, patients treated either with hypo-fractionated radiation monotherapy (60Gy in 25 fractions over 5 weeks), moderately accelerated radiation monotherapy (70Gy in 35 fractions over 6 weeks), or conventional radiation with concurrent chemotherapy, were assessed for locoregional control and distant control at 3 years post treatment. They found locoregional control and distant control to be similar for patients with HPV+ tumors with AJCC 7^th^ edition stage T1-T3N0-N2c disease across the three treatment schedules as well as for patients with HPV- tumors with stage T1-2N0 disease. Patients with more advanced disease demonstrated more clear benefit from concurrent chemoradiation. This study was published during the COVID-19 pandemic with the recommendation that highly impacted treatment facilities adopt the hypo-fractionated approach to conserve healthcare resources and limit patient exposure to the virus.

In the setting of recurrent and metastatic HNSCC, multiple retrospective series have reported good local control and acceptable toxicity with highly hypo-fractionated radiation therapy delivered using an SBRT technique (94–98). These studies have examined heterogeneous patient populations and employed varied dose-fractionation schedules ranging from 13-18Gy in a single fraction (96) to 35-50Gy in 4-6 fractions (97), making comparisons across dose-fractionation schedules challenging. Nevertheless, early evidence of the safety and efficacy of SBRT in this setting have made it an intriguing technique to pair with systemic immunotherapies. Recently, investigators at Memorial Sloan Kettering Cancer Center set out to assess specifically whether employment of SBRT in the setting of recurrent and metastatic HNSCC could stimulate a systemic antineoplastic immune response beyond that generated by immune checkpoint blockade monotherapy (99). The rationale for the study was based on the theory that radiation therapy, and in particular highly hypo-fractionated radiation therapy, can improve the presentation of tumor neoantigens to the immune system, thereby stimulating a more robust antitumor immune response, even in unirradiated lesions (100). This study enrolled 62 patients with recurrent or metastatic HNSCC, and all patients were required to have at least two cancer lesions, one of which could be irradiated, and one of which could be monitored for response by RECIST criteria. 30 patients were randomized to receive nivolumab monotherapy, while 32 received nivolumab plus SBRT to one metastatic site to a dose of 27Gy in 3 fractions. The primary outcome was the overall response rate in the unirradiated lesions. Unfortunately, the study was not able to meet its primary endpoint. There was no statistically significant difference in overall response rate between the two arms (34.5% for nivolumab monotherapy *vs*. 29% for nivolumab plus SBRT, p=0.86). Similarly, there was no statistically significant difference in overall survival, progression-free survival, response duration, or grade 3-5 toxicity between the two arms. The authors concluded that they could find no evidence of synergistic effect between SBRT and immunotherapy in unselected patients with metastatic HNSCC.

Lastly, efforts are underway to determine whether select patients with favorable HNSCC can safely be treated definitively with doses of radiation that are far lower than the current standard of care. The recently reported 30 ROC Trial was a single institution study that investigated the feasibility of using hypoxia imaging to identify patients that could be effectively treated to a dose of 30Gy in 15 fractions rather than the standard 70Gy in 35 fractions (101). In this study, 19 patients with T1-2, N1-2b p16+ cancers of the oropharynx or unknown primary underwent surgical resection of the primary followed by ^18^F-MISO PET to assess oxygenation status of their unresected nodal disease. Patients without pre-treatment hypoxia were assigned to receive chemoradiation to the post-op bed, gross nodal disease, and elective cervical lymphatics to a dose of 30Gy in 15 fractions with two doses of bolus cisplatin or carboplatin/5-FU. Patients with evidence of tumor hypoxia were started with standard of care chemoradiation but could be reassigned to low-dose radiation if gross disease became normoxic within 10 days of starting therapy. All patients who underwent low-dose radiation underwent a selective neck dissection 3-4 months after completing radiotherapy to assess for pathologic response. Ultimately, 15 of 19 patients enrolled were assigned to receive a low dose of radiation. Eleven of 15 had a pathologic complete response on completion neck dissection. Two-year locoregional control was 94.4% (95% CI 84.4-100%) and two-year overall survival was 94.7% (95% CI 85.2-100%). While the results of this study are early, uncontrolled, and applicable only to a highly-select population of patients, they are encouraging in that they represent a willingness to study the employment of novel and less intensive radiation strategies in HNSCC. Whether 30Gy is sufficiently low to reduce the immunosuppressive effects of radiation remains unknown, and additional work will be required to determine whether such a treatment strategy is more compatible with immunotherapy than is conventional radiation.

In summary, despite encouraging evidence of tolerability and safety, and proven efficacy in the recurrent and metastatic setting, clinical trials of checkpoint inhibition have not yet demonstrated efficacy when combined with definitive-intent radiation therapy. The reasons for this remain unclear, however, antagonistic activity from the radiation itself is an intriguing hypothesis. Based upon preclinical principles, theoretical approaches to reducing antagonism between radiation and checkpoint inhibition could potentially include altering the relative timing of delivery of radiation and immunotherapy, hypofractionating radiation therapy to reduce potentially immunosuppressive effects on the tumor immune microenvironment or reducing the volume of elective radiation coverage to promote communication between the primary tumor and the draining lymphatics and promote early antitumor immunity. Clinical evidence for any of these strategies, beyond basic safety and tolerability, remains lacking, however, and careful work will be required to ensure that the proven benefits of conventional radiation therapy are not lost in the effort to increase its synergy with immunotherapy.

### Neoadjuvant

Local therapy, either surgical or radiotherapeutic, is the standard of care strategy for the initial management of non-metastatic SCC for the majority of sites in the head and neck. However, the propensity of these tumors to recur both locoregionally and distantly despite aggressive local therapy, as well as the significant morbidity associated with definitive treatment of locoregionally advanced disease, has made neoadjuvant or induction systemic therapy an attractive treatment approach. The efficacy of induction chemotherapy prior to definitive chemoradiation has been assessed in multiple phase III clinical trials (102). However, with the exception of nasopharyngeal carcinoma (103), improvements in overall survival have not been consistently observed (8, 104–107). Similarly, neoadjuvant chemotherapy prior to definitive surgery has not been shown consistently to improve survival over upfront surgery (55, 94).

The development of checkpoint inhibitors and other novel immunotherapeutic agents has reignited interest in neoadjuvant strategies for locoregionally advanced head and neck cancers. As with chemotherapy, the goals of neoadjuvant immunotherapy include upfront treatment of potential distant sites of microscopic metastatic disease and downstaging of locoregional disease to decrease treatment-associated morbidity while increasing efficacy.

Currently, multiple clinical studies are ongoing to assess the efficacy of immunotherapy in the neoadjuvant setting for HNSCC, and the promising results of several trials have been reported. Early neoadjuvant immunotherapy studies employed cytokine-based therapies combined with immunomodulatory chemotherapy (108, 109). Their results suggested these regimens were tolerable and did not adversely impact subsequent surgery. Tímár et al. found that neoadjuvant immunotherapy altered the ratio of CD4+:CD8+ T cells within the surgically excised tumors (109). Wolf et al. found a potential correlation between the degree of tumor lymphocyte infiltration after neoadjuvant immunotherapy and survival (108). More recent studies have employed checkpoint inhibitors in the neoadjuvant setting. The CIAO trial, the first study to examine ICI for oropharyngeal squamous cell carcinoma (OPC) in the neoadjuvant setting, compared durvalumab *versus* combination durvalumab and tremelimumab in stage II-IVA OPC, finding that safety endpoints were met and that combination therapy did not increase CD8+ T lymphocyte tumor infiltration above durvalumab alone (110). At the ESMO annual conference in 2017, Ferris et al. presented the early results of the CheckMate 358 study of nivolumab in the pre-operative setting for patients with HNSCC (111). Twenty-nine patients with previously untreated, resectable HNSCC of the oral cavity, pharynx, or larynx with at least T1 primary disease and at least N1 nodal disease received nivolumab 250mg on days 1 and 15 and underwent surgery on day 29+/-7. The primary endpoints of the study were safety and a delay >4 weeks for planned surgery. At the time of data lock, four grade 3-4 treatment related adverse events had been reported, and there were no protocol defined surgery delays. CT-defined tumor responses were observed prior to surgery in 11/23 evaluated patients. More recently, Uppaluri et al. published the results of a multicenter phase II study investigating the safety of and pathologic response to pembrolizumab in the neoadjuvant setting (112). Thirty-six patients with stage III-IVb (AJCC 7^th^ ed), HPV-unrelated SCC of the oral cavity, larynx, hypopharynx or oropharynx were administered a single dose of pembrolizumab (200mg) 2-3 weeks prior to surgical resection. Patients with high-risk pathology at surgery, defined as extranodal extension or positive margins, received postoperative chemoradiation and adjuvant pembrolizumab (200mg) every 3 weeks for 6 doses. Low and intermediate risk patients could receive post-operative radiation if indicated but did not receive adjuvant immunotherapy. The co-primary endpoints of the study were the percentage of patients with at least 50% pathologic tumor response at time of surgery and 1-year relapse rate in patients with high-risk pathology compared with a historical control. There were no reported grade 3-4 adverse events associated with treatment and no unexpected surgical delays. Twenty-two percent of patients achieved at least a 50% pathologic tumor response and an additional 22% achieved a 10-50% response. Baseline PD-L1, immune infiltrate, and interferon-gamma activity were associated with achieving a pathologic tumor response. In patients with high-risk pathologic features, the 1-year relapse rate was 16.7%, which was numerically lower than the historical comparator rate of 35%, though not statistically significant. There were no relapses at 1 year in the low-intermediate risk group. Schoenfeld et al. have also recently published results of a phase II study of neoadjuvant checkpoint inhibition prior to surgical resection for HNSCC (113). In this single-center study at the Dana Farber Cancer Institute, twenty-nine patients with at least T2 or node positive SCC of the oral cavity were randomized to receive either two cycles of nivolumab (14 patients) or two cycles of nivolumab and 1 cycle of ipilimumab (15 patients) prior to undergoing surgical resection. Adjuvant radiation or chemoradiation was given according to standard of care based on pathological findings. The coprimary endpoints were safety, including surgical delays, and volumetric response. At 14.2 months median follow up, two grade 3-4 events were observed in the nivolumab arm and five grade 3-4 events in the ipilimumab-nivolumab arm. There was one death that was not thought to be related to the study drugs. There were no unplanned surgical delays. A volumetric response, based on re-staging imaging obtained a median of 14 days after treatment initiation, was observed in 50% of patients in the nivolumab arm *vs*. 53% in the ipilimumab-nivolumab arm. Responses met RECIST criteria in 13% and 38% of patients in each arm, respectively. Clinical to pathological downstaging at the time of surgery occurred in 69% and 53% of patients in each arm, respectively. Intriguingly, post-immunotherapy, pre-surgery PET/CT restaging imaging demonstrate a high rate of increased FDG avidity in cervical lymph nodes that later proved to be pathologically negative, suggesting that immune response could potentially confound interpretation of post-immunotherapy FDG-PET imaging.

At this time, the preponderance of evidence from published studies of checkpoint inhibitors in the neoadjuvant setting suggests that these agents are tolerable, with low rates of severe adverse events, and do not lead to unexpected surgical delays. While clinically meaningful efficacy endpoints remain to be assessed, the clinical and pathological responses observed to date have been encouraging and correlative histopathological and radiological analysis has provided important information for selection of patients for late phase clinical trials. Currently, there are several additional early phase trials underway to further assess the safety and feasibility of checkpoint inhibitors and other immunotherapeutic agents in this setting (**Table 2**). These trials, which feature checkpoint inhibitors delivered as monotherapy or in combination with agonistic immunomodulators or radiation, will provide key insights into the treatment sequences that maximize responses. Of note, there are two open phase III trials examining neoadjuvant immunotherapy in HNSCC. The MK-3475-689 trial (NCT03765918) will assign 704 patients with locoregionally advanced HNSCC to receive upfront surgical resection or neoadjuvant pembrolizumab for two 21-day cycles prior to surgical resection. Patients randomized to the pembrolizumab arm will also receive adjuvant therapy for fifteen 21-day cycles. All patients will receive standard of care adjuvant radiation or chemoradiation based on pathological assessment. The co-primary endpoints of the study will include the proportion of patients with a major pathological response (defined as less than or equal to 10% invasive SCC in the primary specimen) and event free survival for up to 5 years. Similarly, the IMSTAR-HN (NCT03700905) will deliver neoadjuvant immunotherapy prior to surgery with risk adapted adjuvant therapy and maintenance immunotherapy; however, in this study, the investigational drug will be nivolumab with a sub-cohort also receiving ipilimumab in the maintenance phase. The primary endpoint of IMSTAR-HN will be DFS.

**Table 2:** Illustrative listing of clinical trials examining combination immunotherapy in the Neoadjuvant setting for LA-HNSCC.

Trial	Ref	Phase	Study Design	Study Eligibility	Primary Outcome	Status	Estimated Completion
NCT02274155		I	Anti-OX40 x3 (0.4mg/kg 1-3 weeks pre-op) → S → RAA	LA-HNSCC	Safety/Feasibility	Active, Not Reruiting	Oct-22
NCT02488759 (CheckMate 358)	Ferris 2017 ESMO	I/II	Nivo x2 (240mg D1 & D15) → S D30 → RAA	Multiple Cohort (including HPV+ HNSCC)	Objective Response Rate	Active, Not Reruiting	Aug-22
NCT03003637		I/II	Nivo x2 +/- Ipi x1 (240mg and 1mg/kg Wk 1 & 3 pre-op) → S → RAA	LA-HNSCC	Pathologic Treatment Response, Delay to Surgery	Completed	Feb-21
NCT03247712		I/II	Nivo x3 + Xrt x3 (240mg and 8Gy GTV+3mm x3) → S → RAA	LA-HNSCC	Delay to Surgery	Recruiting	Dec-26
NCT02296684	Uppaluri CCR 2020	II	Pembrolizumab x1 (200mg 2-3 weeks pre-op) → S → RAA +/- Pembro x6	HPV- LA-HNSCC; any site	Major Pathologic Treatment Response	Active, Not Reruiting	Dec-21
NCT02919683	Schoenfeld JamaOnc 2020	II	Nivo x2 (3mg/kg Wk1 & Wk 3) or Nivo + Ipi (3mg/kg & 1mg/kg Wk1) → S → RAA	LA-HNSCC; oral cavity	Volumetric Treatment Response	Active, Not Reruiting	Apr-24
NCT03021993		II	Nivo x3 or 4 (3mg/kg D1, 15, 29 +/- 43 for response or stable disease) → S → RAA	LA-HNSCC; oral cavity	Objective Response Rate	Active, Not Reruiting	Dec-21
NCT03721757		II	Nivo x1 (240mg 1-2 weeks pre-op) → S → RAA +/- Nivo x6 (480mg q4wk)	LA-HNSCC; oral cavity	Disease Free Survival	Active, Not Reruiting	Nov-23
NCT02827838		II	Durval x2 (D1 & D14 pre-op) → S → RAA	HNSCC (any stage, surgical candidates)	Immune Biomarkers	Recruiting	May-21
NCT03708224		II	Atezo (840mg total from D1-D15 pre-op) +/- Tiragolumab or Tocilizumab → S → RAA +/- Atezo x12 (1200mg q3 weeks)	LA-HNSCC	CD3 T Cell Tumor Infiltration, R0 Resection Rate	Recruiting	Nov-25
NCT04080804		II	Nivo x1 +/- anti-LAG3 x1 (480mg and 160mg) or Nivo x2 +/- Ipi x1 (240mg and 1mg/kg)	LA-HNSCC	Adverse Events	Recruiting	May-25
NCT03247712	Leidner JITC 2021	Ib	Nivo x3 (240mg q2 wk pre-op) +/- SBRT (GTV; 40Gy x5 fxs or 24Gy x3 fxs or 8Gy x3) → S → RAA +/- Nivo x3	LA-HNSCC	pCR, mPR & clinical to pathologic downstaging	Active, Not Reruiting	Dec-26
NCT03765918 (MK-3475-689)		III	Pembro x2 (200mg D1 & D21) → S → RAA +/- Pembro x6	Multiple Cohort (including LA-HNSCC)	Major Pathologic Treatment Response	Recruiting	Jul-26
NCT03700905 (IMSTAR-HN)		III	Nivo x1 (3mg/kg within 2 weeks pre-op) → S → RAA +/- Nivo x3 +/- Ipi x1 (3mg/kg and 1mg/kg q2 weeks)	LA-HNSCC	Disease Free Survival	Active, Not Reruiting	May-24

## Conclusions/Future Directions

Although immunotherapy is now a mainstay of treatment for recurrent and metastatic HNSCC, the addition of immunotherapy to standard therapies in the curative setting has yet to improve outcomes for patients with locally-advanced disease. The effects of ICI on clinically relevant outcomes in the neoadjuvant setting are still largely unknown, and the negative results of the recent Javelin HN 100 trial inspire several thought-provoking questions about how to optimally combine checkpoint inhibitors with chemoradiation. Excitingly, ongoing work in the preclinical arena is promising and raises the intriguing hypothesis that the efficacy of ICI may be improved by treatment strategies that spare communication between a target tumor and its draining lymphatics. As we look ahead, it will be critical to re-evaluate not only the timing for delivering immunotherapies in relation to standard therapies but also the importance of maintaining the integrity of the tumor-immune-lymphatic axis during immunotherapy. However, as exciting as these hypotheses are, careful work will be required to ensure that the proven benefits of standard therapies are not compromised in the effort to maximize the efficacy of immunotherapy.

## Author Contributions

RS-K and ABS contributed equally to the manuscript, including concept, sourcing literature and writing. WS contributed to sections involving curative-intent immunoradiotherapy trials. AS and LM contributed to concept and editing. EC contributed to all aspects of the manuscript from concept to editing. All authors contributed to the article and approved the submitted version.

## Conflict of Interest

The authors declare that the research was conducted in the absence of any commercial or financial relationships that could be construed as a potential conflict of interest.

## Publisher’s Note

All claims expressed in this article are solely those of the authors and do not necessarily represent those of their affiliated organizations, or those of the publisher, the editors and the reviewers. Any product that may be evaluated in this article, or claim that may be made by its manufacturer, is not guaranteed or endorsed by the publisher.
